# Hydrogen bond dynamics of interfacial water molecules revealed from two-dimensional vibrational sum-frequency generation spectroscopy

**DOI:** 10.1038/s41598-021-81635-4

**Published:** 2021-01-28

**Authors:** Deepak Ojha, Thomas D. Kühne

**Affiliations:** 1 Dynamics of Condensed Matter and Center for Sustainable Systems Design, Chair of Theoretical Chemistry, Paderborn University, Warburger Str. 100, 33098 Paderborn, Germany; 2 Paderborn Center for Parallel Computing and Institute for Lightweight Design, Paderborn University, Warburger Str. 100, 33098 Paderborn, Germany

**Keywords:** Molecular dynamics, Chemical physics

## Abstract

Vibrational sum-frequency generation (vSFG) spectroscopy allows the study of the structure and dynamics of interfacial systems. In the present work, we provide a simple recipe, based on a narrowband IR pump and broadband vSFG probe technique, to computationally obtain the two-dimensional vSFG spectrum of water molecules at the air–water interface. Using this technique, to study the time-dependent spectral evolution of hydrogen-bonded and free water molecules, we demonstrate that at the interface, the vibrational spectral dynamics of the free OH bond is faster than that of the bonded OH mode.

## Introduction

The chemistry of water molecules at the liquid-liquid and liquid–gas interfaces is known to be of high significance^1,2^. The role of interfacial water molecules is also crucial in several chemical processes, like “on-water” catalysis^3^. The microscopic structure and orientation of the interfacial water molecules can be studied using nonlinear spectroscopic techniques, like vibrational sum-frequency generation (vSFG)^4^. When a non-centrosymmetric system is irradiated by consecutive infrared (IR) and visible light pulses and the applied IR pulse resonates with the vibrational frequency of the system, an enhanced vSFG signal is obtained. Moreover, by virtue of quantum-mechanical selection rules of vSFG spectroscopy, the vSFG signal vanishes for centrosymmetric systems^5^. This allows to experimentally distinguish the contribution of surface water molecules from that of the bulk. Conventional vSFG spectroscopy provides a time-averaged microscopic picture of the interfacial molecules^6,7^. By adding a complex IR pulse train before the IR/vis pulse sequence, the technique has been extended to time-resolved analogs of vSFG, i.e. time-resolved vSFG (TR-vSFG) and two-dimensional vSFG (2D-vSFG)^8–16^.

Furthermore, the structure and dynamics of the interfacial water molecules have been extensively investigated using molecular simulations^17–21^. In computational studies, within the linear response regime, the second-order susceptibility ($\chi _{ijk}$) is calculated using the dipole-polarizability ($\mu - \alpha$) cross-correlation function^22–26^. We note here that very long simulation runs are required to obtain a well converged dipole-polarizability cross-correlation function. Moreover, the calculation of the 2D-vSFG spectrum becomes more challenging, as it involves determination of the fourth-order nonlinear response functions based on the fluctuations in transition dipole moment, polarizability and vibrational frequency^27^. In this work, we present a simple computational scheme to obtain a two-dimensional vSFG spectrum of the air–water interface. Furthermore, we analyze the spectral features of the 2D-vSFG corresponding to the free and bonded OH modes.

**Figure 1:**
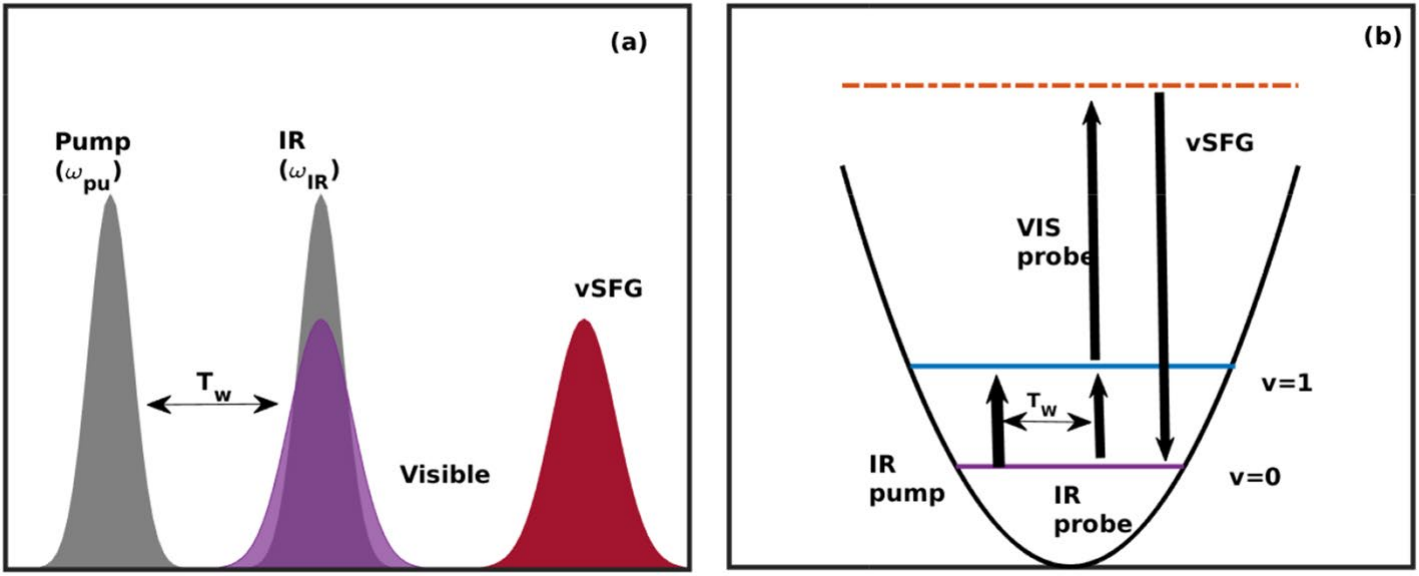
(**a**) Schematic representation of the pulse sequence for time-dependent 2D-vSFG, (**b**) energy level diagram for time-dependent 2D-vSFG excitation.

## Results

The computational scheme implemented in this work has been experimentally demonstrated by Bonn and coworkers^12–16^. We now proceed to elaborate on the method, which is illustrated in Fig. 1a,b. Pump-probe spectroscopy is the simplest case of two-dimensional spectroscopy. However, the interfacial water molecules cannot be selectively analyzed with a broadband IR probe pulse. Therefore, to obtain the surface-specific two dimensional infrared spectrum, the conventional IR probe pulse is replaced by the vSFG probe pulse. The vSFG spectrum is mathematically expressed as^5^

1 $$\begin{aligned} {{\varvec{\chi }}^{\mathbf{2}}_{\mathbf{abc}}} = \int _{0}^{\infty }dt e^{i \omega t} \left\langle {{\dot{{\varvec{\alpha }}}_{\mathbf{ab}}}(0) \cdot {\dot{{{\varvec{\mu }}}_{\mathbf{c}}}}(t)} \right\rangle , \end{aligned}$$

where $\chi ^{2}$ is the second-order susceptibility, $\mu$ is the transition dipole moment and $\alpha$ is the polarizability of the system. The time-resolved vSFG spectrum^28^ can be obtained from the above-mentioned equation without calculating the higher order response functions by explicitly introducing a time parameter $T_{w}$, i.e.

2 $$\begin{aligned} {{\varvec{\chi }}^{\mathbf{2}}_{\mathbf{abc}}}|_{\omega ({t}') = {\omega }'} = \int _{0}^{\infty }dt e^{i \omega t} \left\langle {\dot{{\varvec{\alpha }}}_{\mathbf{ab}}}({t}'+T_{w}) \cdot {\dot{{\varvec{\mu }}}_{\mathbf{c}}}({t}'+T_{w}+t) \right\rangle . \end{aligned}$$

Here, we compute the susceptibility of a vibrational oscillator that is oscillating at vibrational frequency ${\omega }'$ at time ${t}'$. To obtain the 2D-vSFG spectrum of the interfacial water molecules, we applied square IR pulse waves with a FWHM of 50 cm$^{-1}$. The frequency-resolved vSFG spectrum was determined by varying the IR pump pulse from 3300 to 3900 cm$^{-1}$ with an incremental stepsize of 50 cm$^{-1}$. Accordingly, the 2D-vSFG spectrum was obtained by consecutively applying narrowband IR pump pulses via

3 $$\begin{aligned} \sum _{\omega _{i}=3300, 3350 .. \, {\text{cm}}^{-1}}^{ 3900 \, {\text{cm}}^{-1}} {{\varvec{\chi }}^{\mathbf{2}}_{\mathbf{abc}}}|_{\omega ({t}') = {\omega _{i} }} = \int _{0}^{\infty }dt e^{i \omega t} \left\langle {\dot{{\varvec{\alpha }}}_{\mathbf{ab}}}({t}'+T_{w}) \cdot {\dot{{\varvec{\mu }}}_{\mathbf{c}}}({t}'+T_{w}+t) \right\rangle . \end{aligned}$$

**Figure 2:**
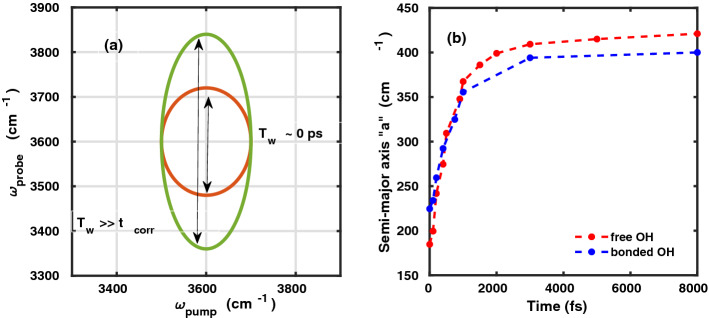
(**a**) 2D-vSFG in inhomogeneous limits, i.e. $T_{w} \ll t_{corr}$, and for homogeneous limits, when $T_{w} \gg t_{corr}$, (**b**) time-dependent evolution of parameter “a” for the free and hydrogen-bonded OH modes.

The purpose of applying a narrowband IR pump is to selectively excite a sub-ensemble of water molecules at the air–water interface. However, to study time-dependent spectral relaxation for the entire width of the stretching frequency, the narrowband IR pump pulse is followed by a broadband vSFG probe pulse, spanning a frequency domain of 3300–3900 cm$^{-1}$. By systematically varying the parameter $T_{w}$, we obtain the time-resolved 2D-vSFG spectrum. For very small values of $T_{w}$, i.e. $T_{w} = 0$, we obtain an inhomogeneously broadened 2D-vSFG spectrum, which is localized around the applied pump frequency. Similarly, for very large values of $T_{w} \gg t_{corr}$, a homogeneously broadened 2D-vSFG spectrum is obtained. The spectral broadening of the 2D-vSFG spectrum for different timescales is schematically illustrated in Fig. 2a. Moving ahead, within our computational scheme, the IR pump pulse is implemented by selective screening of the OH modes vibrating within a given frequency range. Accordingly, instantaneous frequency fluctuations are obtained by using the empirical relationship between the vibrational frequency and the hydrogen bond strength proposed by us in an earlier work^29^:

4 $$\begin{aligned} \Delta E_{D\rightarrow A} \ ({\text{kJ/mol}}) = 0.0392 -150.6 \ \omega \ ({\text{cm}}^{-1}). \end{aligned}$$

**Figure 3:**
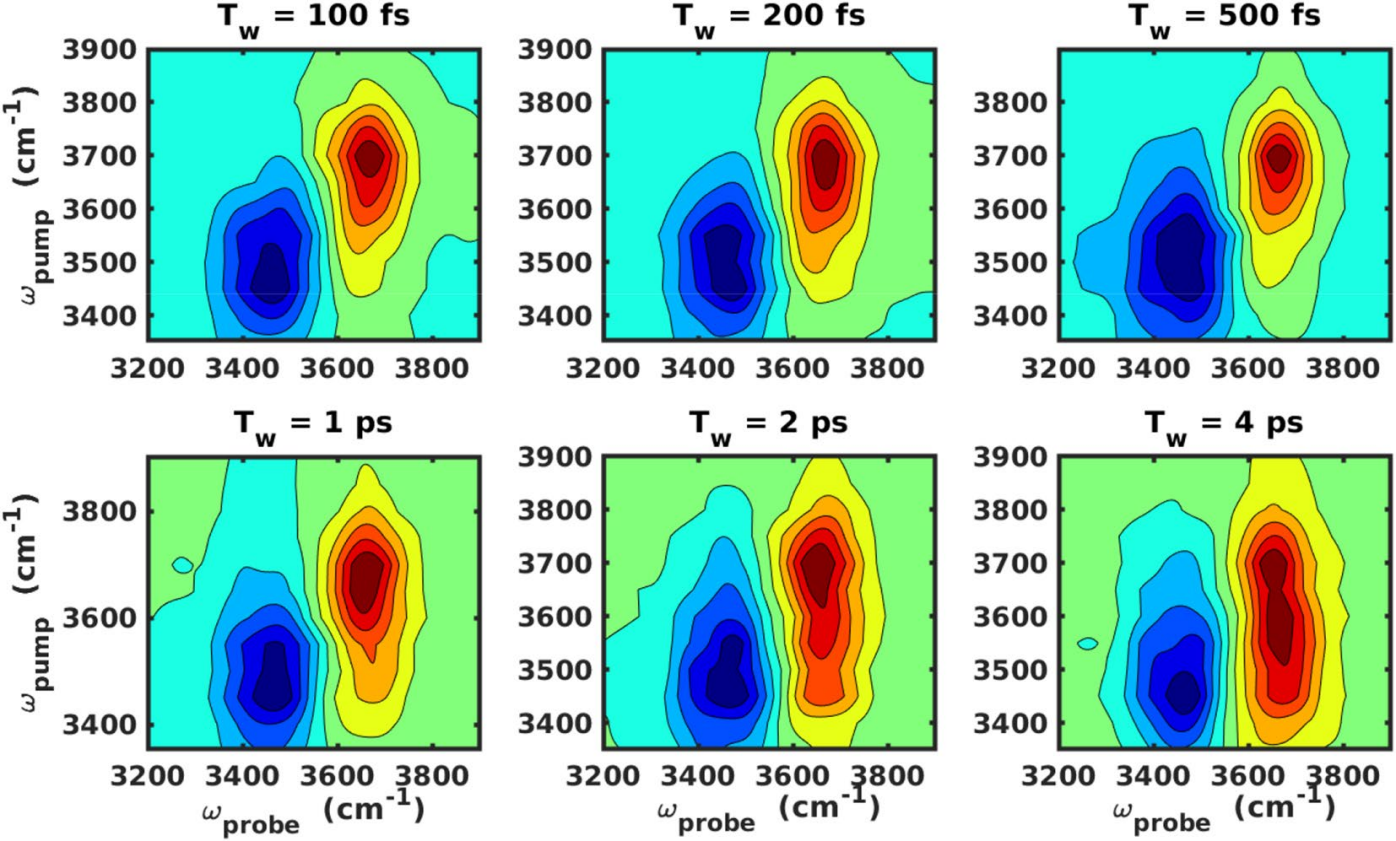
2D-vSFG spectra of the interfacial water molecules as a function of $T_{w}$ waiting times.

The corresponding hydrogen bond strength is obtained from the periodic energy decomposition analysis based on absolutely localized molecular orbitals (ALMO-EDA) calculations^30–32^. The vSFG spectrum is obtained using the surface-specific velocity-velocity correlation function (ssVVCF) method^33,34^. Therein, the *abc* components of the vSFG response function are given by

5 $$\begin{aligned} {{\varvec{\chi }}^{\mathbf{(2)}}_{\mathbf{abc}}} (\omega ) = \left\{ \begin{array}{ll} \dfrac{\mu ^{'}_{str}\alpha ^{'}_{str}}{i\omega ^2} \int ^{\infty }_{0} dt\ e^{-i\omega t} \times \left\langle \sum _{i,j} {\dot{\mathbf{r}}}^{\text {OH}}_{c,j} (0) \dfrac{ {\dot{\mathbf {r}}}^{{\text {OH}}_j} (t) \cdot {\mathbf {r}}^{{\text {OH}}_j} (t) }{{\mathbf {r}^{\text {OH}}_j (t)} } \right\rangle , &{}\quad a = b \\ 0, &{}\quad a\ne b. \end{array}\right. \end{aligned}$$

Here, $r^{OH}_{j}$ and ${\dot{r}}^{OH}_{j}$ refer to the intramolecular distance and velocity, respectively, of a given OH mode. The main advantage of the present ssVVCF method, as compared to other ab-initio based computational approaches that requires the explicit calculation of the polarizability tensor for each configuration of a trajectory^24^, is the much higher computationally efficiency. The latter is due to the fact that in ssVVCF formalism, the dipole moment and polarisability of water molecules are obtained from the fluctuations in velocity and position, which are readily available from ab-initio molecular dynamics (AIMD) simulations at no additional computational cost^35^. By contrast, the employed IR pump-based scheme relies on accurate, yet inexpensive quantum-mechanical ALMO calculations^30–32^. We would like to highlight that the present implementation of an IR pump vSFG probe pulse technique is particularly appealing since alternative computational 2D-vSFG protocols^27^, which involves the direct calculation of the fourth-order susceptibility, are theoretically more rigorous, but rather slow in convergence and thus not feasible within AIMD or path-integral molecular dynamics (PIMD) simulations. Having described the computational narrowband IR-pump and broadband vSFG-probe method, we present, in Fig. 3, the 2D-vSFG spectra of the interfacial water molecules for the waiting times $T_{w} = 0.1, \ 0.2,\ 0.5,\ 1.0, \ 2.0$ and 4.0 ps, respectively.

## Discussion

**Figure 4:**
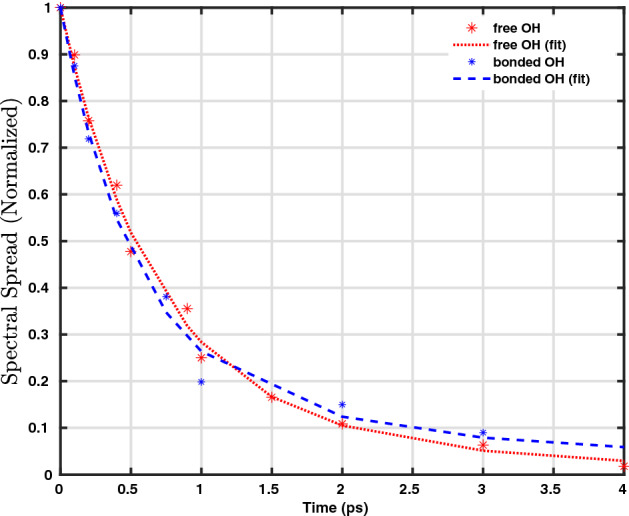
Time-dependent decay of the normalized spectral spread for the free and bonded OH modes of the interfacial molecules. For comparison, the biexponential fit is also shown.

In our computed 2D-vSFG spectrum, we observe two peaks, positioned around 3700 and around 3500 cm$^{-1}$ with respect to the excitation frequency axis. Like in general vSFG, these peaks correspond to the free and hydrogen-bonded OH modes, respectively. The absolute value of the spectral contours refers to the overall orientation of the OH modes with respect to the surface normal. Moreover, the computational IR-pump leads to excitation from the ground state, thus the peaks corresponding to higher level excitations are not observed in our calculations. We note that with an increase in waiting time, the spectral contours corresponding to the free and hydrogen-bonded OH modes tend to spread and cover the entire region of the OH stretching frequency. This spectral spreading is a direct consequence of the hydrogen bond rearrangement. To quantify the hydrogen-bond rearrangement dynamics from our 2D-vSFG spectra, we define a metric denoted as “spectral spread”. For a given waiting time, both of the contours are fitted to an elliptical fitting function. The semi-major axis “a” obtained from the elliptical fitting reflects the extent of vibrational spectral diffusion. The spectral spread is accordingly given as

6 $$\begin{aligned} spectral \ spread(t) = \frac{a_{max} - a(t)}{a_{max}}, \end{aligned}$$

where $a_{max}$ refers to the semi-major axis of the contour for $T_{w} \gg t_{corr}$. The time-dependent evolution of the semi-major axis “a” obtained from the peaks corresponding to the free and OH modes is shown in Fig. 2b. The timescales obtained from the least-squares fit to a bi-exponential function are 1.2 and 1.8 ps, respectively. The time-dependent decay of the normalized spectral spread is shown in Fig. 4. For both, the free and hydrogen-bonded OH modes, the initial fast component is found to be around 300 fs. It is to be noted that the initial fast decay is known to be predominantly associated with the intermolecular librational motion of water molecules^36^. The different timescales obtained for the free and hydrogen-bonded OH modes are consistent with known values from 2D-vSFG or TR-vSFG experiments^12–15^. Having presented our time-resolved 2D-vSFG results, we find it important to emphasize that although the present scheme can be used to study the frequency resolved, time-dependent spectral dynamics of interfacial water, it is based on the computation of second-order susceptibility after an infrared pulse excitation. Accordingly, we circumvent the calculation of the fourth-order susceptibility and corresponding higher order response functions. As a consequence, the cross-peaks corresponding to energy transfer pathways incorporated in the higher response functions cannot be computationally detected. Nevertheless, our simulations predict the timescale of vibrational dephasing for the free OH modes to be around 1.2 ps. In fact, experimental IR-pump vSFG probe measurements have also reported the timescale of 1.2 ps^14^. However, it is necessary to mention that the vibrational dynamics of interfacial water molecules is strongly influenced by the local environment, and, furthermore that the broadening in spectral spread is governed by the dynamics of free and bonded OH molecules.

## Summary and conclusion

Our results support the idea that at the interface, the vibrational spectral dynamics of the free OH bond is faster than that of the bonded OH modes. It is important to note that in our simulations, the effects of inter/intramolecular coupling, vibrational energy transfer and other channels of energy distribution were not taken into account. Nevertheless, the computational approach can be readily applied to study these processes, provided that these effects are incorporated within the simulations or explicitly incorporated in the calculation of nonlinear susceptibility. Moreover, different chemical reactions occurring at the aqueous interface can easily be computationally explored using 2D-vSFG spectroscopy. In addition, the method can be easily extended to study energy transfer between different energy levels by replacing the IR pump with terahertz or X-ray irradiation. In a nutshell, we have demonstrated a computationally efficient and inexpensive approach for obtaining the 2D-vSFG spectrum of interfacial water molecules and applied it to study the hydrogen-bond dynamics of free and hydrogen-bonded water molecules.

## Methods

We have conducted a PIMD simulation in the canonical (NVT) ensemble consisting of 125 water molecules using the flexible q-TIP4P/F water model developed by Habershon and coworkers^37^. Earlier studies have shown that the flexible q-TIP4P/F model is well suited to include nuclear quantum effects (NQE) within structural, dielectric and dynamical, as well as spectroscopic properties of liquid water^37–39^. The air–water interface was generated by creating a rectangular simulation box of 15.5 Å in the x- and y-direction and 46.70 Å in the z-direction. The system was replicated periodically using the minimum image convention. Short-range interactions were truncated at 9 Å and Ewald summation was employed to determine the long-range electrostatic interactions. The ring-polymer contraction scheme with a cutoff value of $\sigma =5$ Å  was used to reduce the computationally expensive part of electrostatic forces calculation to a single Ewald sum^37,40^. While a $p=32$ ring polymer bead was employed, the computationally expensive electrostatic calculations were contracted to the centroid. In contrast to the original PIMD scheme, the partially adiabatic centroid MD technique supports the evaluation of dynamical quantities within the PIMD framework^41^. The effective mass of the ring-polymer beads is adjusted by modifying the elements of the Parrinello–Rahman mass matrix so as to recover the correct dynamics of the centroids and have the integration time-step close to the ionic resonance limit. The temporal evolution of the ring-polymer was performed analytically in the normal mode representation by a multiple time-step algorithm using a discretized time-step of 0.5 fs for the intermolecular and 0.1 fs for the intramolecular interactions^42^. Furthermore, ALMO-EDA calculations^30–32^ were performed for all configurations along the trajectory, which were separated by 10 fs, using the CP2K^43^ code. Interfacial water molecules were obtained using the identification of truly interfacial molecules (ITIM) algorithm^44^.

## Data Availability

The data generated and analyzed during the current study are available from the corresponding author on reasonable request.
